# Editorial: Computational and Experimental Approaches in Exploring the Role of Genetics and Genomics in Multifactorial Diseases

**DOI:** 10.3389/fgene.2022.873069

**Published:** 2022-03-15

**Authors:** Duc-Hau Le, Quang-Huy Nguyen, Lan T. M. Dao

**Affiliations:** ^1^ School of Computer Science and Engineering, Thuyloi University, Hanoi, Vietnam; ^2^ Vinmec Research Institute of Stem Cell and Gene Technology, Hanoi, Vietnam

**Keywords:** multifactorial diseases, genomics, personalized medicine, sequencing, computational approaches, experimental approaches

**FIGURE 1:**
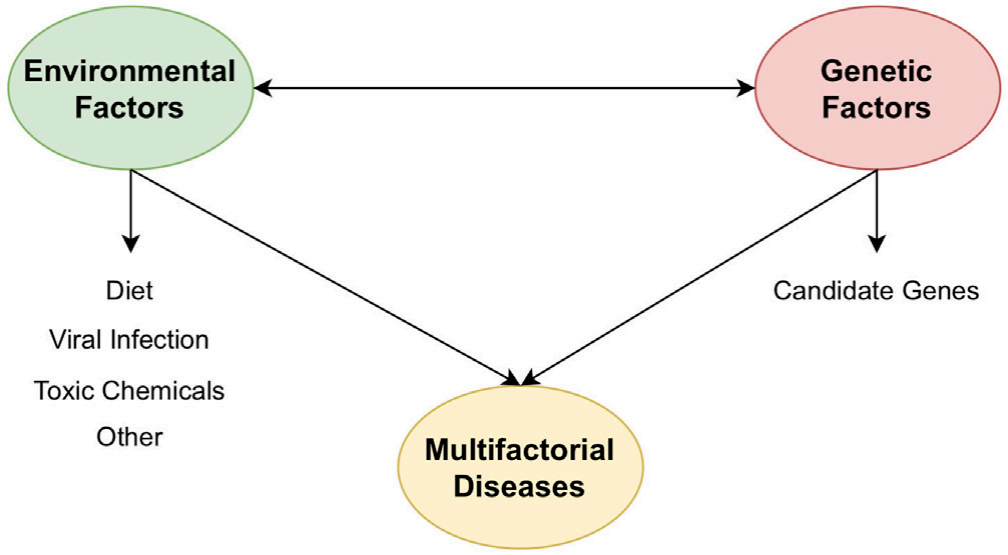
The combination of environmental factors and genetic factors is the main culprit which takes responsible for causing multifactorial diseases.

Multifactorial diseases are ones caused by multiple genetic disorders in conjunction with lifestyle and environmental factors (Figure 1). Although multifactorial diseases often cluster in families, they do not have a clear pattern of inheritance. Besides, the genetic variants associated with the complex disease are often common polymorphisms; therefore, pinpointing and analysis of disease-related genes to unravel disease mechanisms are a must. For these reasons, it is still difficult to study and has left many unanswered questions of multifactorial diseases regarding their etiology. The introduction of next-generation sequencing technologies observed so far has supported us to dissect different multifactorial diseases such as diabetes, cardiovascular diseases, cancer worldwide. We have observed great technological progress in genomics and computing in the last decade, enhancing our understanding of human genetics and diseases. Researchers can now analyze massive quantities of genomic data, by computational and experimental approaches means, to shed light on the pathogenesis of complex diseases.

Nevertheless, there is a pressing need for high-quality investigations (e.g, leveraging the power of integration of datasets, proposing state-of-the-art approaches, etc.) to further explore multifactorial diseases with complex pathogenesis since they are caused by numerous different factors like several variants in different genes. To date, various approaches have improved understanding of how genetics contribute to the development of diseases and the interaction of other factors that constitute diseases. With massive genetic and genomic data generated by next-generation sequencing technologies, the combination of computational and experimental approaches could potentially provide the tools needed to answer some fundamental questions and provide much-needed conclusions.

This research topic of Frontiers in Genetics serves to collect novel cutting-edge computational and experimental methods helping to dive deeper into the underlying principle of genetics and genomics in multifactorial diseases with the emphasis of raising awareness of the role of genetics and genomics in multifactorial diseases. As a result, this can support us to answer unsolved questions such as what is the relative contribution of the genetic, epigenetic, microbiome, and environmental factors? How can genomics data be used to tackle big unanswered questions; such as, what causes the variation in phenotypes, disease susceptibility, and drug responses? From that, this topic makes a call for the submissions related to, but not limited to, the following topics:

• High throughput methods based on genomic, epigenomic, transcriptomic, proteomic approaches in different models to identify molecular profiles associated with multifactorial diseases;
• Identification of novel genomic variations associated with the multifactorial disease;
• Combination of different approaches to identify the mechanism underlying the complex phenotypes in multifactorial diseases;
• Assessment of gene function using genome editing tools;
• Integrating genomics into medicine. Development of personalized medicine based on the genetic data of each individual;
• Decoding multifactorial phenotypes;
• Large-scale genome sequencing data associated with multifactorial diseases.

This research topic received lots of submissions and there were five exciting works accepted for publication. Firstly, Poon and Chen sought to determine the impact of two types of complex disease, including cerebrovascular disease and major depression, on transcriptomes of non-diseased human tissues and investigated whether inflammation has any effects on cerebrovascular events. They showed there were statistically significant associations between the two complex diseases above and the gene expression of non-diseased tissues. Furthermore, the systemic and long-lasting effects of cerebrovascular events were potentially in relation to inflammation. Secondly, Shi et al. analyzed the associations of the expression profiles of long non-coding RNA (ncRNAs), micro RNA (miRNAs) and mRNA with rare-earth pneumoconiosis, the main occupational disease of rare earth exposed workers. They discovered the differential expression of 125 lncRNAs, 5 miRNAs, and 82 mRNAs in the plasma of patients with REP, and showed the relationship between the differential expressions of ncRNA and the response of cells to lncRNA acting as a sponge of miRNA to regulate the target gene in the competitive endogenous RNA networks. Thirdly, Weizhou Guo et al. proposed a new multimodal data fusion model named MAFN for survival prediction of breast cancer subjects by integration of multiple omics datasets like gene expression, copy number alteration, and clinical data, and demonstrated its superiority over other state-of-the-art methods. Fourthly, Yeuni Yu et al. performed an integrated -omics analysis and concluded that SOCS3 was a crucial biomarker for glioma and that SOCS3 was related to the proliferation of neuronal tissue. Lastly, Rahu Sikander et al. developed a novel convolutional neural network-based method to predict the amino acid sequences of enzymes and non-enzymes using both protein geometric structures and protein sequences. The method outperformed other cutting-edge tools which often used structural information or amino acid sequences alone. In summary, this topic recruited some in-depth computational and experimental approaches that dissect the fundamental principles of the role of genetics and genomics in multifactorial diseases.

